# A Rare Disease in Adult: Langerhans Cell Histiocytosis

**DOI:** 10.4021/wjon663w

**Published:** 2013-07-15

**Authors:** Ilhami Berber, Mehmet Ali Erkurt, Irfan Kuku, Mustafa Koroglu, Emin Kaya, Serkan Unlu

**Affiliations:** a Department of Hematology, Inonu University Faculty of Medicine, Malatya, Turkey; b Department of Radiology, Inonu University Faculty of Medicine, Malatya, Turkey

**Keywords:** Langerhans cell, Histiocytosis, Adult

## Abstract

Langerhans cell histiocytosis is a rare histiocytic disorder and has been diagnosed in all age groups, but is most common in children. This disease is very rare in adults. We presented a patient who was 62 years old man diagnosed langerhans cell histiocytosis.

## Introduction

Langerhans cell histiocytosis (LCH) is histiocytic disorder. This disease is most commonly characterized by single or multiple osteolytic bone lesions demonstrating infiltration with or without histiocytic infiltration of lymph nodes, liver, skin, lungs, spleen, bone marrow, or nervous system.

LCH is a rare histiocytic disorder. LCH has been diagnosed in all age groups, but is most common in children. The incidence appears one to two cases per million adults [1].

The generic term “histiocyte” refers to several types of cells, including Langerhans cells, monocytes/macrophages, and dermal/interstitial dendritic cells. These cells into the following three categories: dendritic cell disorders, macrophage-related disorders and malignant histiocytic disorders. LCH is member of dendritic cell disorders [2].

The clinical presentation of patients with LCH varies depending upon the sites and extent of involvement. The disease is limited to one organ system in approximately 55 percent of patients, the others have multisystemic disease bone (77 percent), skin (39 percent), lymph nodes (19 percent), liver (16 percent), spleen (13 percent), oral mucosa (13 percent), lung (10 percent), central nervous system (6 percent) [3].

LCH has been diagnosis with biopsy specimens. It is shown mono or multinucleated Langerhans cells, histiocytes, and eosinophils. The presence of Birbeck granules on electron microscopic examination or of antigenic markers that react with CD1a glycoprotein and the cytoplasmic protein S100 were detected by immunostain. Erdheim-Chester disease, juvenile xanthogranuloma, multiple myeloma, hemophagocytic lymphohistiocytosis, Rosai-Dorfman disease (sinus histiocytosis with massive lymphadenopathy) are thought in differential diagnosis [4].

In initial treatment, the patients are eveluate which risk group stratification; single or multisystem LCH. Single system LCH; patients have no systemic symptoms. Unifocal or multifocal involvement can be found in one of organs/systems (bone, skin, lymph node, lungs, central nervous system). Multisystem LCH; two or more organs/systems are involved with or without involvement of hematopoietic system, liver, and/or spleen and denote a worse prognosis [3].

There have not been clinical trials to inform practitioners of the optimal therapy of adult patients. The combination of vinblastine plus prednisolone is the most commonly used induction regimen and is administered over six weeks, as follows. Cladribine, bisphosphonates, tyrosine kinase inhibitors, other treatments (metotrexsat, mercaptopurine) are be used in recurrence LCH. The role of allogeneic hematopoietic cell transplantation in LCH has not been directly studied [5, 6].

## Case Report

**Figure 1:**
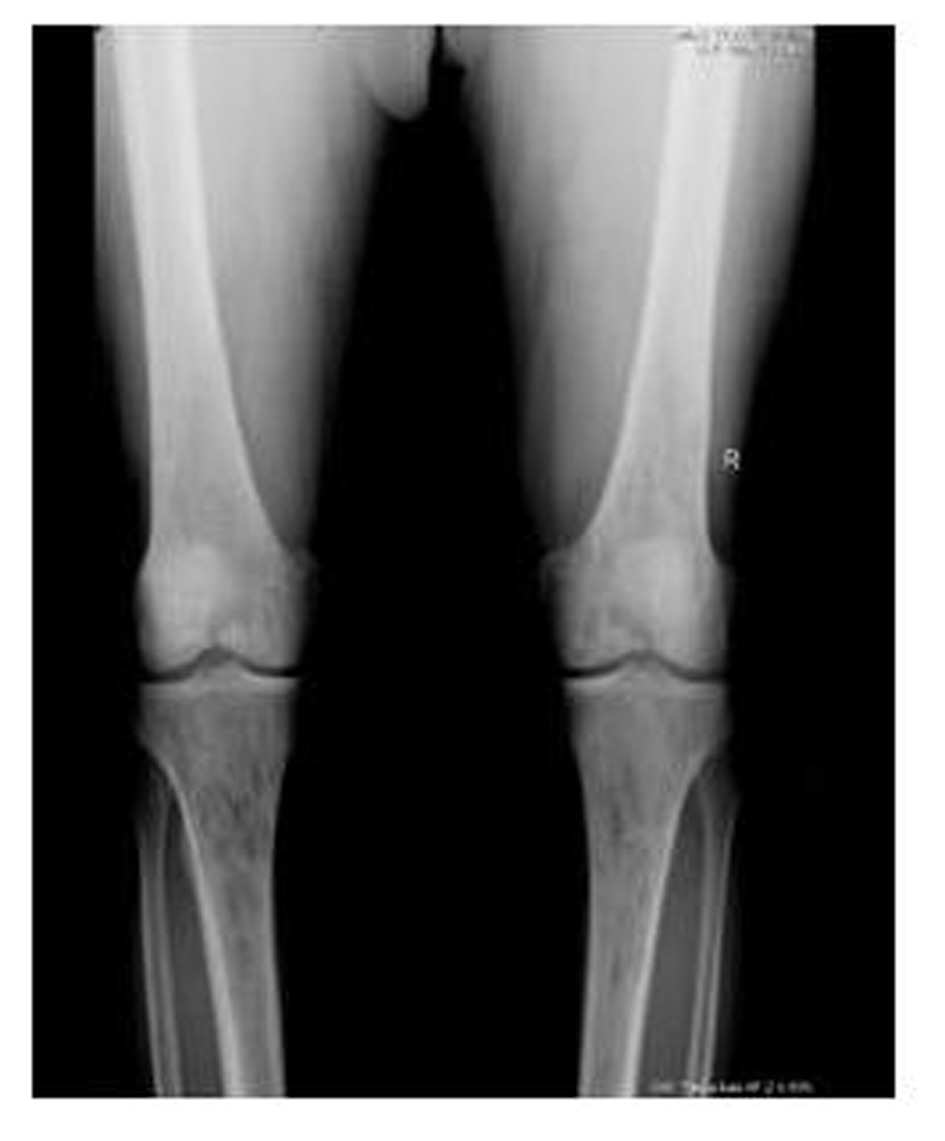
Radiolucent and osteolytic lesions with irregular margins were seen in bone structures of both knee joint’s epiphysis, metaphysis and diaphysis.

**Figure 2:**
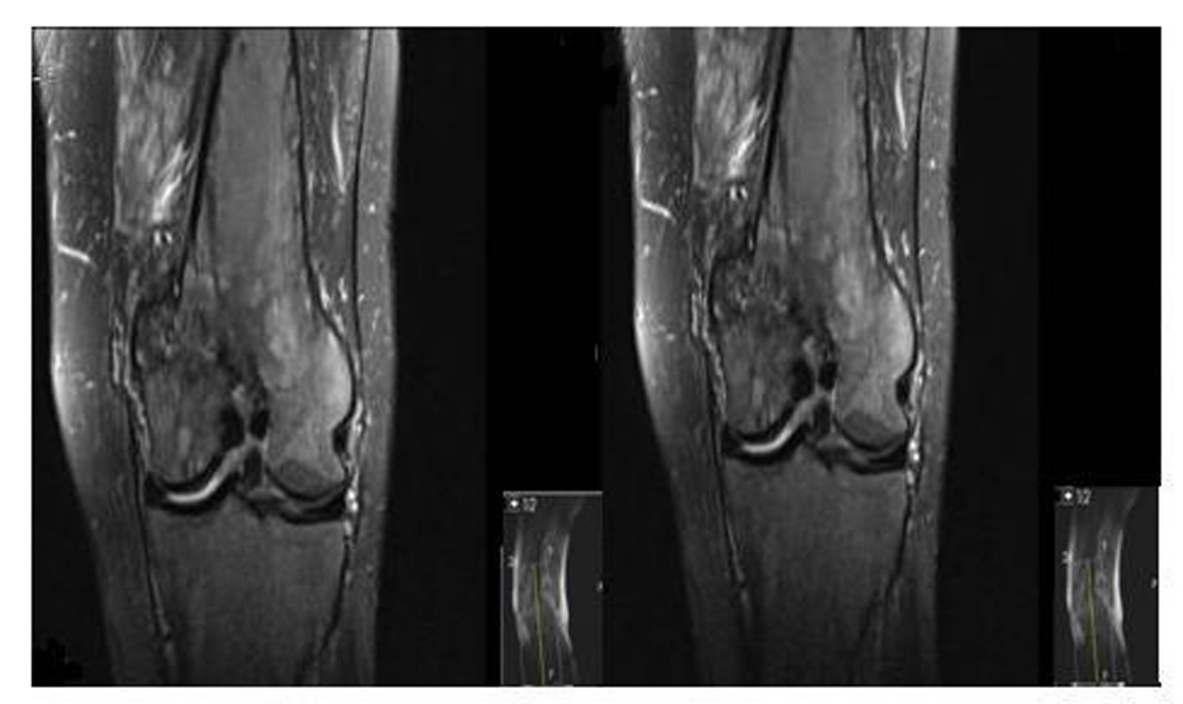
Pathological signal changes observed in both of knee joint.

**Figure 3:**
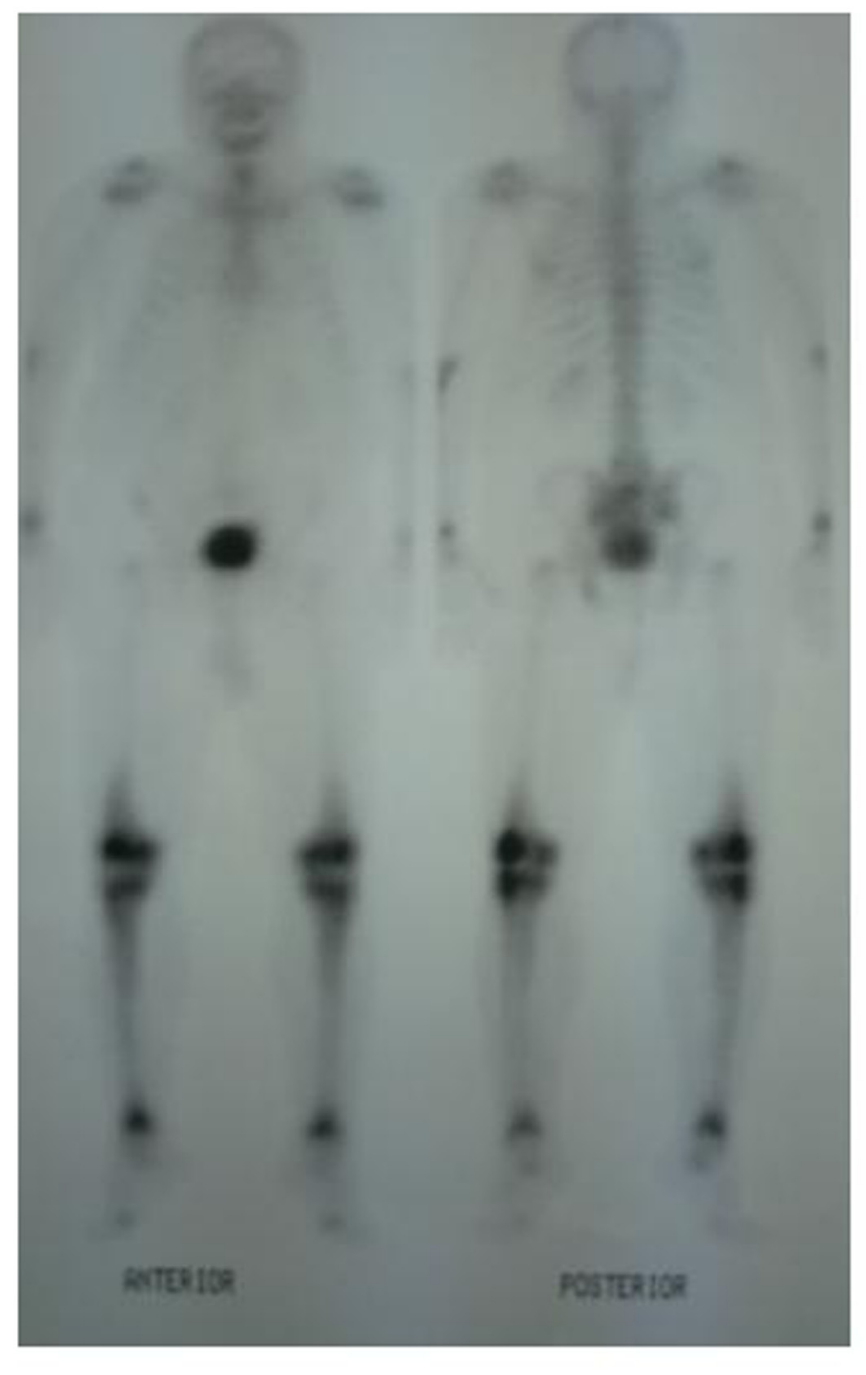
Increased osteoblastic activity was detected in bilateral proximal and distal tibia by whole-body bone scintigraphy.

A 62-year-old male patient with knee pain, fatigue, night sweats and was admitted to our clinic. In patient history, central diabetes insipidus was diagnosed 3 months ago by nephrology clinic and vasopressin treatment was started. In laboratory examination, leukocyte counts 8.600/µL, hemoglobin level 12.6 g/dL, mean corpuscular volume 75 fL, platelet count 466.000/µL, blood urea nitrogen 8 mg/dL, creatinine 0.7 mg/dL, total protein 6.2 g/dL, albumin 2.8 g/dL, lactate dehydrogenase 184 U/L, sodium: 142 mmol/L, potassium 4.3 mmol/L, sedimentation 31 mm/h, C-reactive protein 6.17 mg/dL, thyroid stimulating hormone 2.1 µIU/mL. Peripheral blood revealed 65% neutrophils, 30% lymphocytes, 5% monocytes, cluster-platelet aggregates and normal red blood cell morphology. Immunoglobulin levels and immune fixation electrophoresis test were normal. Tomography of the abdomen and thorax was normal. Bone marrow examination was normocellular and no neoplastic infiltration. Jac-2, BCR-ABL were negative and karyotype was normal. Lytic lesions were detected in lower extremity radiographs (Fig. 1). Imaging signal changes were detected in bilateral distal part of the femur and proximal of tibia with by magnetic resonance (Fig. 2). Increased osteoblastic activity was detected in bilateral proximal and distal tibia by whole-body bone scintigraphy (Fig. 3). Biopsies were taken from tibia and was reported Langerhans cell histiocytosis (CD 1a (+), CD68 (+), Langerin (+), S-100 fokal (+)). Vinblastine 6 mg/m^2^ (6 weeks, 1 time per week), prednisolone 40 mg/m^2^/day (every day for 4 weeks) therapy was started. Zoledronic acid 4 mg/day treatment was started as once a month. After vinblastine, prednisolone therapy, patient was evaluated for response and progression was detected. Cladribine 0.14 mg/kg/day (for 5 days, 28 days) treatment was designed for 6 cure, 6 cure cladribine treatment was completed and patient was evaluated with knee MRI and whole-body bone scintigraphy again. These imaging techniques performed continuous regression.

## Discussion

LCH is in a rare disease and most common in children. The incidence appears one to two cases per million in adults. LCH is most often female dominant and average 43.5 years old. Our patient was 62 years old man, 62 years is unacceptable old for this disease.

Patients are usually fever, night sweats, weight loss and nonspecific symptoms such as fatigue and organ involvement may present with complaints [7]. Our patient admitted to our clinic with fever, fatigue and generalize bone pain.

LCH is divided two classes according to extend of involvement; single or multisystem LCH [3]. Our patient has involvement of pituitary and extensive bone morrow and evelauted as multisystem disease.

Central Diabetes insipidus is the most frequent endocrine abnormality in LCH. Diabetes insipidus typically presents with polyuria, nocturia, polydipsia. Other endocrine anomalies of LCH include hypogonadism, growth failure, abnormalities of glucose metabolism and an enlarged thyroid gland [8]. Polyuria, nocturia, polydipsia revealed in our patient. Pituitary magnetic resonance performed 3 mm microadenoma and he was diagnosed central diabetes insipidus. He was treated with vasopressin treatment. Other endocrine abnormalities were not detected in our patient.

Bone involvement identifies in the most of patients with LCH. The patient may complain of pain in a localized area of bone and a raised, soft, tender spot are detected in examination. Radiologic studies typically demonstrate a lytic, “punched out” appearance. Soft tissue mass sometimes accompanies to lytic lesion. In a study, jaw (30 percent), skull (21 percent), vertebra (13 percent), pelvis (13 percent), extremity (17 percent), and rib (6 percent) were primary sites of bone involvement in adults [1]. Our patient admitted our clinic with knee pain. Lytic lesions were detected in lower extremity radiographs. Imaging signal changes were detected in bilateral distal part of the femur and proximal of tibia with by magnetic resonance and whole-body bone scintigraphy revealed increased osteoblastic activity in bilateral proximal and distal tibia. We thought multiple myelom as differential diagnosis, but immunoglobulin levels, immune fixation electrophoresis test were normal and there was no increased plasma cell in bone marrow examination.

The combination of central diabetes insipidus (DI) and lytic lesion are thought LCH and performed biopsy from an osteolytic bone. Pathologic evaluation of involved tissue should be performed for diagnosed LCH from a biopsy of an osteolytic bone lesion or skin lesion. Positive immunohistochemical staining for CD1a and CD207 or the identification of Birbeck granules by electron microscopy should be detected for diagnosed LCH. CD 1a (+), CD68 (+), Langerin (+), S-100 fokal (+) were detected in our case. We could not able to evaluate CD207 and Birbeck granules because of lack of technical capacity.

In differential diagnosis, LCH must be distinguished histologically and immunophenotypically from other histiocytic and dendritic cell disorders, metastatic solid or hematopoietic neoplasms, hemophagocytic lymphohistiocytic and macrophage activation syndromes [4].

In initial treatment, vinblastine plus prednisolone therapy is suggested for patients [5]. We applied vinblastine plus prednisolone therapy to our patient for six-week course. The posttreatment imaging study of choice is exactly unknown. Imaging studies performed at the time of diagnosis, and depends upon the site(s) of involvement. Magnetic resonance and positron emission tomography can be performed in posttreatment evaluation [9].

Disease response is categorized to four groups; No evidence of active disease, complete resolution of all signs and symptoms, continuous regression; signs and/or symptoms are less prominent with no new lesions, Stable disease; persistence of signs and/or symptoms, Progressive disease; progression of signs/symptoms and/or appearance of new signs/symptoms [10]. After 6 weeks of vinblastine plus prednisolone treatment, knee MRI was applied and progression was seen in our patient.

There is a little information regarding the treatment of refractory LCH. For patients with low risk LCH who relapse less than 12 months from the end of the original vinblastine plus prednisolone treatment, single agent Cladribine (for six months therapy) results in responses in approximately 60 percent [11]. Our patient was applied 6 cure cladribine treatment. Knee MRI, whole-body bone scintigraphy were applied to patient again for response evaluation and continuous regression was detected after 6 cure cladribine therapy.

In conclusion, when an elderly patient admitted with litic lesion, langerhans cell histiocytosis must be kept despite it is a child disease.
